# Taking Care of Forced Migrants Together: Strengths and Weaknesses of Interorganizational Work from the Perspective of Social Workers

**DOI:** 10.3390/ijerph20021371

**Published:** 2023-01-12

**Authors:** Amalia De Leo, Giulia D’Adamo, Carlotta Morozzi, Caterina Gozzoli

**Affiliations:** 1 Department of Psychology, Catholic University, 20123 Milan, Italy; 2 Department of Medicine and Surgery, University of Parma, 43126 Parma, Italy

**Keywords:** inter-organizational work, forced migrants’ care, reception centers, social workers, services network

## Abstract

The reception and taking care of forced migrants with mental health issues is undoubtedly a very complex task. The literature shows that reception systems are characterized by a high level of fragmentation due to poor collaboration among services that are required to respond to complex and multidimensional needs brought by forced migrants. Starting from the need to deepen what elements support or hinder the implementation of the services’ networks for the care and management of forced migrants, qualitative research was conducted within a constructivist paradigm. As the literature reveals a lack of studies considering the perspective of practitioners, we decided to explore the representations of professionals working with migrants in northern Italy in four different service areas (health and psychological well-being, reception, family, and legal areas). A total of 24 professionals (13 F and 11 M) with an average age of 42.4 years were involved in four focus groups according to the four areas. A paper–pencil content analysis was conducted following IPA guidelines. The results show strengths, weaknesses, and elements of improvement for service network implementation. The needs of forced migrants seem to be effectively met only through a service network that takes shape in the interweaving of social, organizational, group, and individual levels.

## 1. Introduction

Cooperation between organizations and the sharing of specific skills between professionals has become an ever more pressing necessity in the reception and care of forced migrants [1]. Network work is defined as the set of interventions designed to connect people, groups, and institutions through connections and meaningful interpersonal and cross-functional relationships. The main purpose of a network of services for the reception and care of forced migrants is to provide an effective and satisfactory response to the needs brought by the beneficiaries. The economic, social and political crisis that involved Europe in recent years has led to the restructuring of the welfare state, which has been characterized by processes of outsourcing to social cooperatives and other private entities of services historically guaranteed by public institutions [2,3,4]. Katz and Nowak (2018) describe this phenomenon as the ‘new localism’, referring to radical shifts in the location of power [3]. Contemporary policies to manage migration and reception flows of ‘asylum seekers,’ and ‘refugees’ are located precisely within these processes of welfare reform, and as a result, they have been invested with the affirmation of the principle of subsidiarity [5] which has led to a widespread decentralization of services; in particular, the governance and care of migrants’ lives have shifted from being a national to a regional/municipal responsibility. This has had positive consequences for the system, but it has also brought criticalities to light that had not been noted in the past. More specifically, while the local definition and management of social issues, especially if they are complex and sensitive, make it possible to implement relevant projects, they also contribute to defining a very varied and fragmented overall situation in which it can be difficult to orient oneself and identify common, or at least shareable, practices among the different realities operating in the territory. In this respect, the first element of difficulty concerns the realization of efficient cooperation between the central and the local governments [6]. Indeed, the coexistence of various regulations, not always consistent with each other, has resulted in inconsistent interpretations between one service to another and in the implementation of different intervention practices [6]. In addition, concerning migration, actors’ views at national and local levels can be divergent [7,8,9,10]. A reasonable solution would be to use different network actors in new configurations to fill gaps and change local practices by collecting local, regional and national partners in horizontal networks [11,12,13,14].

The literature highlighted the full complexity of the needs of those who forcibly migrate due to extreme circumstances, such as persecutions or contexts of generalized violence brought by war or civil conflict, and with an extremely fragile, if not entirely nonexistent, migration project [15,16]. The almost-total lack of legal access to protection exposes these people to a very dangerous migratory path marked by exploitation, sexual violence, malnutrition, inability to be cared for, psychophysical humiliation, detention and refoulement [17,18]. In addition, people who emigrate face a range of post-migration difficulties when they arrive in Europe; their vulnerable condition [16] exposes them to further traumas related to inadequate reception conditions and the real risk of falling into severe marginalization [19,20].

Considering this situation, services must provide an immediate and flexible response to ensure the protection of refugees and human rights. Housing, financial support for food, and a medical care plan are the first basic needs that must be met [21]. Moreover, it is widely recognized that language plays a key role in the process of social inclusion [22]; for this reason, refugees must learn the local language and cultural aspects and have legal services and employment assistance to better settle in the host communities [21,23]. At the same time, it is important to set up, alongside medical care services, those aimed at taking care of the mental health of refugees since mental health across immigrants is mainly affected by stressful and traumatic experiences that take place before, during and after the migration [24,25,26]. According to Stockermer et al. (2020), following the “European Refugee Crisis” of 2015 [27], the number of mental health care needs to be increased in host countries [28]; as a consequence, public and private mental health care systems were immediately overburdened, and often the emergency department was the only access to mental health care system [29,30]. Relating to mental health care services for migrants in Paris, Tortelli et al. (2020) [31]—in line with other European countries—found that first, mental health specialists believe that they do not possess enough knowledge of migrant psychology/psychopathology, and this excludes migrants from professional care delivered by the public system [32]; second, due to restrictive migration policies, the inconsistent access to welfare benefits or the absence of a stable housing made access and continuity of mental health services even more difficult [33].

Looking at the Italian context, various research on the topic shows that the cultural paradigm of emergency remains the model of reference [34] even though the phenomenon of migration towards Europe is frequent, unstoppable and destined to last. Territorial services struggle to offer adequate responses, and they are not always proactive towards other services that deal with the care and reception of migrants. In many territorial contexts, there is a lack of professionals with adequate specific skills, and where available, they are too limited in number to respond to the existing need [35,36,37]. Reflecting on their professional practices, operators underline the presence of ‘black holes’ concerning time, space, relationship and back-office management. These shadow areas often do not allow for the appropriate strategies to be identified and implemented to welcome and care for migrants.

From the above situation, it emerges that the reception systems present a high level of fragmentation due to the request of different needs. Complex and multidimensional needs, such as those expressed by forced migrants, require equally complex and multidimensional responses: this is why, almost inevitably, taking care of this population ends up highlighting all the fragilities due to the poor integration between social services and health services that, despite the progress made, still characterizes many territories [1]. Therefore, even if cooperation between different professional figures, and especially the networking of specific competencies, would represent a crucial improvement of migration policies, the aim of synergetic networking of services is not easily achievable, as it requires considerable energy and resources, both economic and organizational and professional [8,9,10].

Furthermore, even though the literature review shows an accurate analysis of the needs of those who benefit from these services [21,22] and highlights the need to implement multilevel governance for complex issues facing societies (poverty, crime, health promotion, economic development, natural disaster, education, health care reform) [1], there are no studies that consider the networks of services for the reception and mental health care of forced migrants, and fewer still consider the professionals’ perspective.

For this reason, we decided to implement a qualitative study to answer the following question: what are the key elements that allow the different professionals and local institutions to connect and cooperate to effectively respond to the complex and individual needs of forced migrants?

More precisely, we conducted a study that considers the point of view of workers who work with the management and mental health care of forced migrants daily, exploring:

- Weaknesses of the network of services;
- Strengths of the network of services;
- Areas for improvement of the network.

## 2. Materials and Methods

### 2.1. Aims and Scopes

Starting with the desire to deeply understand what elements favor or hinder the process of taking care and the synergistic management of forced migrants, qualitative research was carried out within a constructivist paradigm [38] to explore the representations of professionals working in northern Italy in four different service areas: health and psychological well-being, reception, family and legal. Generally, these are the four main areas of services that interact and work together in taking care of forced migrants with mental health issues of the second level of reception. Table 1 shows the types of organizations and specific tasks of each area. The urban area we analyzed has always been an important destination for the mobility path of economic migrants, and it represents a significant place where temporary reception experiences for asylum seekers are put in place. The territory is characterized by a strong historical presence of services [39], and for this reason, it could be considered a privilege to investigate which synergic actions support and promote reception and which care systems provide positive outcomes.

### 2.2. Participants

A total of 24 professionals were involved, including 13 females and 11 males, with an average age of 42.4, belonging to 15 public and private social organizations. Specifically, four were psychotherapists, four were legal practitioners, four were coordinators/managers, four were cultural mediators, two were psychiatrists, two were social workers, three were practitioners, and one was a psychologist—all of which belonged to the four areas of intervention we considered (Table 2). The inclusion criteria considered for the professionals were that (1) they provided assistance to forced migrants or worked in one of the four areas considered; (2) they worked in northern Italy; and (3) they voluntarily participated in the study. Instead, the main exclusion criterion was the inability to participate in the focus groups during the study period. The people intercepted were evenly distributed in each of the four areas. We believe we achieved a sufficient level of data saturation with our sample.

### 2.3. Measures

The focus group is a particular form of multiple interviewees that allows the collection of valuable material on the point of interest. Focus groups are characterized by specific group interactions that allow for the topic of interest to be treated with greater complexity and, at the same time, enrich the participants’ points of view [40]. With such an instrument, therefore, the aim was to enhance the crucial role of the experience of practitioners by making explicit their professional practices referring to network work [41]. In fact, the presence at the same table of different professionals also belonging to different realities made it possible to bring to the table crucial points and critical issues that would not have emerged with the individual interview alone as the outcome of the group interaction. Indeed, the continuous comparison between social actors makes it possible to clarify individual positions and compare them with those of others, according to a process of sharing and comparing [42] that leads to the definition and explication of subjective meanings. This way, it is possible to collect the many meanings behind the same term or expression. Moreover, an instrument that would consider group dynamics seems to be particularly appropriate in reflecting the functioning of multiprofessional networks. These dynamics do not occur in a two-person interview, where the interaction, while present, is undoubtedly between asymmetrical actors. Furthermore, in an interview, the interaction is only linear, whereas, in the group, it is reticular, which broadens the stimuli. In other words, the focus group proves to be the instrument most faithful to the setting and working reality of network professionals. In our case, we opted for online Focus Groups because, due to the COVID-19 pandemic, strict physical distancing protocols to protect public health were still in place during data collection.

Online focus groups have emerged as a leading research option for collecting primary qualitative data during the COVID-19 pandemic; evidence suggests that with careful adaptations, conducting groups online can replicate and be comparable to conducting groups in person [43,44]. In particular, to improve its effectiveness online, we made some adaptations: set up smaller groups to encourage interaction among participants, reduced the length of the session (about 2 h) since people’s ability to concentrate and stay engaged is more limited in the online format, and made some arrangements to encourage everyone’s participation (for example, we asked them to keep the video camera on throughout the session).

### 2.4. Methods and Procedure

For each of the four involved areas, a focus group (for a total of four) was conducted with the aim of exploring whether and which professional practices practitioners act in their daily work regarding networking. Because the Interpretive Phenomenological Approach is used to study the meanings experience hold for participants [45,46,47], the focus group schedule included questions about personal representations and feelings about the strengths and weaknesses of interorganizational work (“what is your professional role?”; “can you tell us what are the main struggles in the networking with other practitioners/organizations”; “can you tell us what are the main resource aspects in networking with other practitioners/organizations”; “can you tell us about an experience where you think networking worked very well in the process of taking care of forced migrants or vice versa worked poorly?”). All focus groups were conducted through the Zoom platform. Before each group session, a consent form and an anonymous sociodemographic form with a brief description of the research project was sent to each participant; they were asked to complete and return it by the day of the meeting. The focus groups lasted an average of two hours and were conducted by one researcher. All sessions were recorded with the consent of the participants. The research was approved by the Ethics Committee on Psychological Research, Department of Psychology, Catholic University of the Sacred Heart.

### 2.5. Data Analysis

Subsequently, the focus groups were fully transcribed, and a paper–paper content analysis was conducted anonymously [48] following the procedure of the Interpretative Phenomenological Approach, IPA [49]. IPA aims to understand the experiences and explore the process of constructing meaning that individuals use to understand the subjective perspective and consider the sociocultural context in the data interpretation process [45,50,51]. During the text analysis, a process of decomposition and segmentation of the focus groups was performed by reducing them to extracts to which appropriate categories were assigned to define their meaning. By gradually integrating and selecting categories that were thought to be related by similarity of meaning, more general concepts were gradually constructed. Through an inductive approach, interpretive categories were created by integrating and selecting concepts that included them and suggested typical uniformities of behavior or context. Two researchers reviewed the interview materials, summarized and extracted meaningful statements, and formulated the present themes. Conflicting opinions on the contents of a theme were discussed and resolved. The interpretive categories thus constructed allowed the formulation of a generalizable interpretation that will be reported below. Specifically, the results will focus on the categories that emerged by revolving them around the axis of fatigues and resources with respect to the theme of synergistic networking. Finally, insights for improvement that professionals have reported will be reported.

## 3. Results

The results of the study will be reported below. Table 3 shows a summary of the categories that emerged from the data analysis.

### 3.1. Weaknesses of the Network of Services

#### 3.1.1. Hostile Social Context

The operators who participated in this research reported that their job was hindered by the increasing political and media hostility. One of the main elements of effort in doing their job was the emergence of a climate of political and media hostility towards the world of welcoming services and the professionals involved in caring for forced migrants. In recent years, since the approval of the Security Decree, the activation of political security dynamics tending toward ‘criminalization’ led to a progressive closure towards migrants with consequent repercussions on operators’ work.

*“In the last few years, due to changes in politics, I realized how much hostility there is towards reception services and towards specific professional categories. As a professional who’s working in this area, I perceived a greater hostility from the citizens. I believe that this is one of the consequences of the economic crisis, and it is also due to political events which spread a different reception culture.”*

(Operator in Health and Psychological Wellness Area)

*“…with the Security Decree in 2018, which became law, the fundamental institution of humanitarian protection came to a standstill.”*

(Operator in Legal Area)

This hostility promoted a climate of skepticism and suspicion towards migrants and, therefore, towards services and operators themselves. Furthermore, the right to access the refugee centers became very hard for asylum seekers (e.g., the abrogation of humanitarian protection), and this led to a considerable increase in the number of irregular migrants who are not involved in any service network.

*“In my opinion, a constant problem is the difficulty of approaching all irregular migrants who have not been accepted into dedicated facilities because they do not have the requirements for or any kind of international protection.”*

(Operator in Health and Psychological Wellness Area)

#### 3.1.2. Structural Lack of Resources

At an operational level, there were cuts in costs for the management of reception centers and a reduction in the already insufficient resources available. These cuts have further drained the network of services. Speaking of which, operators highlighted a lack of ‘structural’ resources, especially in institutions such as traditional psychiatry, which has been supporting demand for a long time.

*“From my point of view, there is still a great hole in traditional psychiatry institutions. Bringing new cases to a structure already saturated, it seems to me that the attention and desire of the most valid and available doctor are linked to the resources that are in the structure itself.”*

(Operator in Reception Area)

*“There are particular situations that require specific tools to be solved, which we do not currently have.”*

(Operator in Legal Area)

The bigger lack is perceived in public services because the hostile social and political context has led operators to feel unsupported and discouraged in their work. This phenomenon has entailed both a growing delegation to the private sector and some services taking care of needs that are not specific to their particular organizational assignment.

*“Because most of the migrants do not have a family, they look for social structures; the social contribution is typically asked to the third sector and not to local government. The inclination to delegate to the private sector is more and more present, and we put all our strength into play, but it is necessary a structural reform by the region of Lombardy and the Ministry of Health to overcome these structural fragilities in the public sector.”*

(Operator in Reception Area)

#### 3.1.3. Negative Emotions

In this context, operators do not feel safe, especially in the management of cases of severe mental unease. Indeed, migrants’ stories are often characterized by rage and discouragement; this had inevitable consequences on the mood of the operators, who reported feeling scared and isolated.

*“It is everyone’s problem: operators, institutions and migrants. Because that rage, that aggressiveness and that discouragement feeling could affect the operators’ integrity and the institutions themselves. This is the biggest fear that lingers and belongs to our project.”*

(Operator in Reception Area)

#### 3.1.4. Organizational and Management Difficulties

Furthermore, overloaded schedules, a burdened climate at work, and a general perception of fatigue represent an important organizational limit that hinders networking and synergies with other actors. At a procedural level, it is emphasized that a network collaboration between operators is not systematically structured, but it relies on individual initiative. Indeed, the single one may be motivated to collaborate and interact with other institutions on the territory.

*“As far as we are concerned, we wedge interventions in an overloaded schedule that provides for something else. We are aware that this is a limit, considering that each intervention requires the networking of several actors: the beneficiary, us and the mediator. We perceive a bit of stress in doing these things; on the other hand, we need more time and a less rigorous agenda.”*

(Operator in Health and Psychological Wellness Area)

*“Networking has always been difficult and is unfortunately promoted very little. It is easier not to do because it takes a lot of time and effort to deal with. Moreover, teamwork requires collaboration with people with whom we may have almost nothing in common.”*

(Operator in Reception Area)

#### 3.1.5. Institutional Rigidity

According to operators, some institutions are very closed (e.g., the school), and this attitude makes networking even more difficult. In addition, the static nature of some actors in perpetuating their thinking model, focused on a single working standard, makes collaboration strenuous. The operators report a lack of adequate skills as well for networking and valid tools at their disposal.

*“I noticed this kind of closure on the part of some institution. There was a tendency to use one’s own standard, one’s own model of thinking. An ethnological approach with more understanding of the person’s story is surely more difficult. It requires time and could be not in all operators’ expertise, although it would be in everyone’s best intentions.”*

(Operator in Family Area)

*“Having poor training is a weakness, for sure. Also, the lack of an intercultural aptitude is a gap.”*

(Operator in Legal Area)

*“I have to say that there is an inadequate formation and general disorientation on the theme. In my opinion, the asylum seekers have always been managed as a separate universe.”*

(Operator in Family Area)

#### 3.1.6. Insufficient Communication between Services and Not-Clear-Roles

Operators denounced a lack of clarity in the definition of the different actors’ roles. There is so much confusion that there is an overlap of expertise and limited coordination between both horizontal services and institutions. In some services, operators declared they felt of being less considered and recognized than other specialists in the territory, with whom they have occasional and unstable rapports. Especially, a general sense of isolation and fatigue emerged in the interaction between the legal area services and institutions considered to have a low level of intercultural awareness (e.g., police headquarter and prefecture (In Italy, the Immigration Desk is established at each Prefecture—Territorial Office of the Government; the Ministry of the Interior, the Ministry of Labor and Social Policies and the Police Headquarters are part of it)). This limited predisposition has consequences on the well-being of the beneficiaries.

*“First of all, we lack the right tools. We are service, a reality among a lot of services in the Brescia area. We should define the roles first. There is a lack of coordination and communication between horizontal networks, which should coordinate better to be able to manage cases properly and find the right solution.”*

(Operator in Legal Area)

*“Many services consider little the legal area. […] very often when I deal with social workers, and they pass me a case, they don’t even know which residence permit people have if they have one.”*

(Operator in Legal Area)

#### 3.1.7. Fragmented Post-Migratory Paths

The fragmentation of services inevitably has repercussions on migrants who “ramble” from one service to another; the person is, in fact, forced to tell his or her story again at each step. This process can harm both the migrant by causing psychological suffering and the institutions that lack a comprehensive and cohesive story. During this transition, the migrant must narrate and re-narrate his own story, with the risk of causing further suffering and losing some parts (fragmentation of the stories). This makes the reception context a place of conflict: migrants are feeling abandoned by the institutions, and they harbor feelings of anger and frustration towards this fallacious system. In other words, a specific number of arbitrary and institutional procedures seem to characterize the reception and migrants’ care processes. These services are managing the paradoxes of a system that, on the one hand, claims to accept and, on the other, reiterates the sufferance itself.

*“One limitation, something that I always felt was very important, is the fact that there are so many actors within the network. The beneficiaries, then, are forced to repeat many times something that is always new to us. Listening to their story is obviously necessary to be able to do our work, but for them, it is the telling of something that I feel is very violent [for them] every time.”*

(Operator in Health and Psychological Wellness Area)

*“Feelings of anger are present. I am convinced that we are working in a place of conflict;: we are aware that the reception process will never be linear and easy. It has consequences for us, the beneficiaries, on the territory where the conflict takes place. If we cannot manage this kind of conflict, we will never be able to understand how to elaborate positively. In addition to the conflict, we are harboring drastic feelings, such as anger. These days I am taking care of certain people with huge anger towards us, but they should address this feeling towards institutions that are responsible for this anger.”*

(Operator in Reception Area)

#### 3.1.8. Totalizing Institutions

Migrants perceive the reception centers as places of transit because they lack long-term planning and they focus on short-term projects instead. Although, the trauma and past efforts have different recovery times than those offered by these centres. Indeed, also operators have a hard time helping the migrants to build projects that make them autonomous.

*“There is no space for these young people to reveal a situation of discomfort in a SPRAR (the system of reception and integration promoted by the Ministry of the Interior and the local authorities) project. The vulnerable kids we deal with need their time for some situations to emerge. History tells us that some steps are fundamental; indeed, the most serious situations emerged after a period of tranquility when their emotions and efforts have had a safe context to be told.”*

(Operator in Reception Area)

*“Most of them live in these structures as a place of transit, where they wait for specific answers (i.e., their documents). When these answers come, they will make their own decisions without investing a lot.”*

(Operator in Reception Area)

*“The SPRAR project was thought of as a long-term project, and it should offer specific tools to address people’s autonomy. In that way, migrants should be able to assimilate into the host society at work and socioeconomic levels. Indeed, after a SPRAR project dedicated to mental needs, there should be something else that is not there.”*

(Operator in Legal Area)

### 3.2. Strengths of the Network of Services

#### 3.2.1. Mental Health Care

Despite the presence of known difficulties, the operators also reported positive aspects about the different topics investigated. The attention of the operators toward the mental health of migrants is a fundamental resource in the identification of mental illnesses. Compared to the past, there is a greater competence in managing psychological well-being. On a practical level, there is a clear definition of the objectives in the first phase of Reception: to take charge of the person and support him along a path of listening and care.

*“In my opinion, it is not that clients are changed. What has really changed is how we accept their psychic suffering in the different areas of response.”*

(Operator in Health and Psychological Wellness Area)

*“Recently, we have been working in teams in a more qualitative way. Lately, we are hosting fewer people, but the care for them is 360°. We are trying to address the person’s suffering from multiple perspectives. In fact, there are educators and professionals who activate individual pathways for inclusion, such as alphabetization, both for migrants inside and outside of refugee centers.”*

(Operator in Health and Psychological Wellness Area)

#### 3.2.2. Professionals’ Interpersonal Relationships

Professionals consider the collaboration and intertwining of several professional skills necessary to cope with problems that a single operator would not be able to solve alone. Cooperation would guarantee an integrated approach to the person’s needs, from psychological aspects to social integration, from medical assistance to job placement. Interviews highlighted, in fact, that there are good interpersonal relationships between professionals, which would favor continuity in the care pathway. However, since these relationships depend exclusively on the intentionality of individuals—as they are not formalized—they cannot always guarantee an effective process of synergic collaboration.

*“As a service, being in touch with these realities is fundamental. It exists from our point of view, which is [that of the] legal operators’. We are in contact with the structures that deal with mental health, and it is useful to understand the work practices that we are supporting. Then the exchange of view could be both unidirectional and bidirectional.”*

(Operator in Legal Area)

*“What has changed in the approach is the necessity to collaborate and intertwine more professionalism to face bureaucratic problems. Indeed, a single CPS (mental health center) social worker, a single outpatient doctor or even just a single institution alone could not manage face up to.”*

(Operator in Health and Psychological Wellness Area)

*“Collaborating with the START project has made it possible to have alternative points of view, different interpretations and having home care support both for these situations and interviews, which are conducted by the psychologist in charge. This collaboration has allowed us to work in synergy and, in my opinion, respond fair enough to the needs of families. These are families that come here anyway and have no support other than that of Reception centers. They do not have a great possibility to ask people for help except for a few friends and relatives.”*

(Operator in Family Area)

#### 3.2.3. Relationship with Beneficiaries

The relationship with clients seems to represent an important resource for professionals. From the interviews, a strong ideal drive emerges, supporting the motivation of professionals and acting as a protective factor against the fatigue that working with migrants brings with it [37]. It seems that the network gaps are often balanced by the positive effect generated by authentic relationships that professionals are able to establish with beneficiaries. In many cases, in fact, professionals are the only points of reference for migrants on the territory even after they leave refugee centers. This professional reports how, in her experience, working with migrant families has been particularly rewarding because of the opportunity to create emotional bonds, especially with minors.

*“We specialized in working with these families. I have to say that working with asylum seekers is very satisfying […] Although there are difficulties in working with families, the fact that there are minors gives the idea that there is also a sort of future, a hope for the future for these people. Also, for the operators, it is not a bad thing because it gives us the energy and strength to continue. It is also nice to see children being born; indeed, it is very nice.”*

(Operator in Family Area)

#### 3.2.4. Acknowledgment and Trust among Services

Finally, an aspect that seems to emerge, especially in the legal area, concerns the feeling of acknowledgment and trust between the actors in the system. According to professionals, despite the many legislative changes, there was no distrust of operators towards their work. This professionalism/skill was also recognized by some ‘external’ interlocutors such as the police headquarters and prefecture.

*“I have to say that I have never felt mistrust from other professionals for the work we do at the legal support office, in the sense that in any case, we have tried to help beneficiaries to get residence permits despite legislative changes […]. So, I must say that the attitude of people has actually been very helpful; I have always found people who were very trusting of the legal accompaniment that we have tried to provide even in this transitional period.”*

(Operator in Legal Area)

### 3.3. Possibility of Improvement in the Quality of the Network

#### 3.3.1. Need to Formalize the Network

All operators share a common need: having greater collaborations between different institutions due to specific organizational devices. Since the moments of transition from one service to another are incredibly important, the main objective would be to define a synergic network in continuous communication, which acts promptly in order not to aggravate already overt conditions of fragility. Therefore, it would be desirable to have spaces and coordination themes that allow sharing, comparison and consideration around a theme. Structured management of services would be more holistic and effective.

*“If you do an interview with a beneficiary, you have to consider at least two or three networks because working with migrants always requires connection and synergy with colleagues. So, services need to formalize this requirement and have moments of coordination; it would help us a lot.”*

(Operator in Health and Psychological Wellness Area)

*“…periodic meetings are defined with the main subjects we interface with: police headquarters, prefecture, and commission. It does not mean that we must see each other every month, it could also be twice a year, but it must be a place where all the problems and the possible solutions are put on the table. They should not fall on deaf ears! So, we should meet again and see how it goes on and what progress has been made. In this sense, it would be interesting if psychiatry institutions will take part in this meeting because mental illness is an ever-growing element, and we already talk about this at our ‘Periferie della cura’ (Peripheries of care) meeting (“Peripheries of care” is an interinstitutional board involving different local actors (Province and Municipality of Brescia, Provincial Coordination of Italian Reception System projects, Spedali Civili Brescia hospital) that deal with the reception and care of forced migrants. The main objective of the board is to investigate and compare practices and theories of care of the forced migrant)”*

(Operator in Legal Area)

*“In my opinion, it is very interesting to think about networking among the different realities that deal with forced migrants, especially in a municipality like Brescia that also has SPRAR. The municipality of Brescia manages different sectors: the SPRAR is the inclusion sector, while the social services sector that deals with the territory are precisely another sector, so let’s say that there is not a constant flow of communication constant between the two sectors and instead, it would be very important that there is”*

(Operator in Family Area)

#### 3.3.2. Development of New Tools

In order to protect the migrant, the establishment of a professional figure as a case manager was proposed. The case manager supports the person from the first time they are taken in charge and throughout all the processes. Alternatively, a more realistic and sustainable solution could be to establish a single folder that summarizes the path of the person so that they are not forced to narrate and re-narrate their own story and the trauma experienced. This folder would also make it possible to keep the story intact without losing relevant elements. It would be easier for the migrant to be taken in charge through the various services. In addition, to encourage a more effective communication process between the beneficiary and the various services in charge, there is a need to have the constant presence of figures that facilitate dialogue in different contexts, such as the cultural mediators.

*“Keeping the presence of a constant mediator and not changing him: […] often the mediator is the person who already knows the patient’s story. The mediator helps the person to ‘re-narrate’ his or her story, to remember certain very intense themes of great suffering.”*

(Operator in Health and Psychological Wellness Area)

*“The narration, the re-narration and the ulterior narration of my life, of my discomfort, of my story, of my multifigure needs, goes in parallel with the operator’s need that the migrant narrates, re-narrates, re-tells to each institution what has happened and what’s happening. So, I don’t know if it is practicable or possible, but I think that it would be innovative if these people were given either a case manager or a single folder that collects all the documentation. In this way, whoever intervenes would be updated at any time, and the migrant would be less scared of not re-living everything emotionally again.”*

(Operator in Health and Psychological Wellness Area)

*“So, the need to find new procedures, which had not been thought of before because there was no need for them to exist, leads us to somehow be more creative and find original methods. These methods should be recognized and approved by those who coordinate the institutions.”*

(Operator in Health and Psychological Wellness Area)

#### 3.3.3. Need to Train and Inform

The current and constantly changing social scenario requires professionals with complex and ever-changing skills. Therefore, it is necessary for the operators to have the opportunity to update and deal with new knowledge, discoveries and working methods. Accordingly, there was a proposal to establish an institutional place, a sort of research and training center, for example, where it would be possible to channel knowledge and develop and learn new skills. One professional proposes to establish an ethnocratic center whose primary aim is to include in its therapeutic exercise the treatment of victims of war, terrorism, misery and consequent migration. Ethnoclinical practice considers the individual in his or her richness and diversity in relation to ethnic, religious and political groups. This center would become the focal point of a network that allows professionals not to find themselves isolated, alone, and with few operational, informational, technical, as well as often economic resources, helping to circulate and transmit the operational and clinical knowledge that is gradually acquired in clinical practice.

*“So, from my point of view, we really should open a school, a university space, we’re thinking about these topics finds its institutional place where being inserted. Being able to build a thought that is periodically confronted with all mental approaches would lead to the determination of consequent practices. […] we must also consider the cultural approach.”*

(Operator in Reception Area)

*“There is the idea of setting up an ethnoclinical center, which first and foremost is concerned with training those who work in the public and also those who work in the so-called private sector. […] I would like to think that we can build such a pathway […] a center where we can train and share procedures, projects, practices, and above all help set up to shared thinking.”*

(Operator in Reception Area)

*“Everyone’s role […] is also to spread competence, and likewise, the role of the local social service should be to get information and be involved. […] the goal must be to create a competent community in this field so that they also are on the territory.”*

(Operator in Family Area)

#### 3.3.4. Promote a Culture of Hospitality

Moreover, the need to involve the territory and to promote a ‘Culture of Hospitality’ has emerged. The aim is to spread and raise awareness among citizens on the work done by the services and on the hospitality dimension. From this point of view, the surrounding community becomes a very precious and fundamental ally for structuring truly inclusive paths, especially when the migrants are outside the services networking. This kind of work can be done on multiple levels and in various contexts, such as in schools and universities.

*“We need to find a system to make ourselves promoters, not so much in a specific and specialized, or super-specialized, environment, where we only go to refine things. But we should create a basic resonance that, in the long run, could also generate and that could also be proactive for the single user. We need to become promoters of a territorial culture a little more detailed, concrete and informed about what we do and what our beneficiaries require.”*

(Operator in Health and Psychological Wellness Area)

*“I think being able to work on these themes as well makes the SPRAR project interventions in schools very valuable. When students from middle and high school hear the stories and testimonies experience a little bit firsthand what we are talking about, even before they go to college. That is, this human reality that is not so far away and not so strange.”*

(Operator in Health and Psychological Wellness Area)

*“…because the more information is disseminated, the more effective the service is also for social network and integration of migrants.”*

(Operator in Legal Area)

## 4. Discussion

The research objective was to explore the views of professionals from various services regarding elements of effectiveness, critical issues and aspects of improvement in the network of treatment services for asylum seekers.

**Figure 1:**
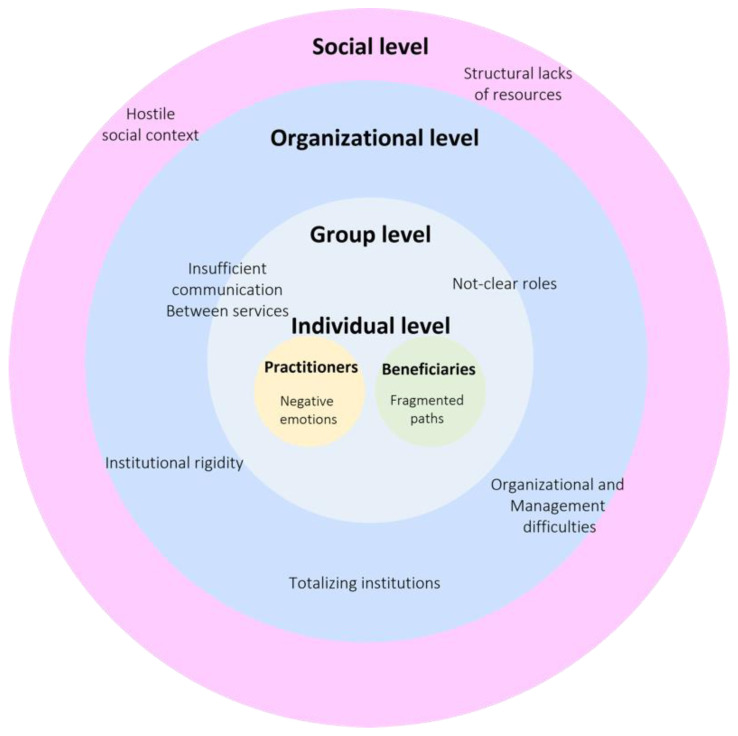
Graphical representation of the service network.

We can say that the functioning of the network of services for the reception of forced migrants is a complex and articulated process in which elements of various levels intertwine themselves: (1) social level (hostile social context, structural lack of resources); (2) organizational level (institutional rigidity, organizational and management difficulties, totalizing institutions); (3) group level (insufficient communication between services, not-clear roles); (4) individual level (negative emotions for workers and fragmented paths for migrants) (Figure 1).

From a social point of view, research participants identified a political situation somewhat hostile to the reception of migrants [52] as a result of some recent legislation (i.e., Security Decree n. 113/2018) and with a clear regional diversity in the management of forced migrants; a situation that fostered a fragmented nature of care services and an absence of common practices in reception procedures [7,16]. In fact, one aspect of great difficulty concerns the implementation of good coordination between central and local governments, which in turn is part of an ambiguous and poorly standardized regulatory framework [6]. The absence of a formalized network results in contacts between institutions only based on interpersonal relationships and informal arrangements (organizational level). The main insights that emerged from the professionals’ words mainly concern the struggles in implementing solid cooperation between services in the care of migrants. The “black holes” that emerge on every operational level (time management, space management, relationship management, back-office management) often do not enable the necessary setting to identify and implement the appropriate strategies for welcoming and caring for migrants. The general perception of providers is one of fatigue in managing and supporting migrants; the operator, in fact, does not feel protected in the complexity of his role (individual operators’ level), as he or she cannot rely on a structured system but only on his/her own goodwill or that of colleagues [53].

Another element of difficulty is the lack of preparation and training by networking; in fact, there is frequently overlapping expertise and poor coordination, both between horizontal services and between institutions (interorganizational level). Beneficiaries are “bounced” from one service to another, making it hard for operators to have a defined picture and history of the person with whom they are interacting. In fact, reception centers and various services are perceived by migrants as temporary places since these institutions favor short-term projects rather than long-term planning. This contributes to the fragmentation of post-migration pathways that do not support the psychological well-being of migrants [34], indeed in some cases; they may cause the reactivation of traumas [54,55] or the creation of new ones in the new context that does not know how to alleviate their difficulties (individual level) [56]. From an organizational point of view, moreover, it emerges that services appear as burdensome and bureaucratic, where it is the user who must adapt to them and not vice versa. A perceived lack of competence in managing the mental suffering of migrants they have to support and care for also emerges from the words of the practitioners [32].

Starting from the analysis of the points of fatigue in the network of care services, the operators identified some aspects to focus on in order to improve the cooperation between services. First, it is essential to reshape the role of each professional—regardless of his/her specificities—who must move from a logic of mere implementation of individual projects to one of cooperation and integrated management of beneficiaries. To do this, it is necessary to develop structured communication skills among service providers so that the fragmentation that occurs in the current network will be as limited as possible. This idea can be translated at an operational level into the development of new tools such as a shared medical record, the establishment of a new role as a case manager, or the implementation of a platform on which it is possible to share information about the personal history in order to connect the different services. In addition, to foster more effective communication processes between users and services in charge, it was raised importance to provide a constant presence of professionals who can facilitate interlocution, such as cultural mediators. Also emerged the need to create an “Integrated Map” of local resources (associations, prefecture, social services, legal offices, etc.) to be shared among the service network to facilitate the orientation of professionals and migrants between different realities.

In order to develop communication between services in the network, a strong demand for training has emerged, driven by the necessity to share practices, dialogue spaces, and reflections and to promote the development of critical thinking.

Occasional networking projects between services have already been carried out on this territory. Still, once concluded, they were unable to give continuity to the intersystem work that was implemented, partly because of professional turnover. It is, therefore, necessary to set up stable working groups to take charge of these transitions in order not to dissipate the know-how acquired. This could be realized by establishing a shared research center (i.e., an ethnoclinic center) that could become an institutional reference point to avoid the dispersion of knowledge and experience.

More generally, at the national level, it would be necessary to promote a “Culture of Reception”, shared with the community, and moved by a more synergistic logic of taking charge, so that the migrant, once the reception process is over, can fully integrate into the new social context.

The present study is not free from limitations. First, we involved only some specific categories of professionals; therefore, future research could deepen the perspective of other professionals, such as cultural mediators, who have a privileged view of the services since they are in contact with several different operators at the same time and have a deeper and more continuous contact with the beneficiaries. In addition, in this study, we did not consider the migrants’ point of view; for this reason, it would also be important to explore their experience as users of the services network. As a final aspect, we highlight the possibility of investigating social networks and social support of the migrants and their role in preventing mental disorders; moreover, it would also be important to investigate whether there is partial overlap with the services network and whether there can be a process of strengthening mutual relations between the two networks.

## 5. Conclusions

The consequences of the economic, social and cultural crisis led to a reduction in resources, thereby amplifying the sense of powerlessness regarding the capacity of the various services to operate, including those dedicated to the care and reception of forced migrants. Moreover, the progressive decentralization of services—which have shifted from a national to a territorial/regional government—together with a sociopolitical context that, over the years, has become increasingly hostile towards migrants, has contributed to a varied and fragmented scenario that cannot always cope with the complexity of migrant needs. Our study highlighted that, intervening in, for and with the network becomes the privileged sphere of intervention as we start from the awareness that a single service cannot respond to the complexity of the demand of forced migrants who, as we have seen, can only find an answer in the intertwining of social, organizational, group and individual dimensions. Professionals, in this perspective, have an intersystemic role since, by working on a case-by-case basis, they can gradually build up an in-depth knowledge of the person and his/her problems. This allows them to “grasp how the beneficiary’s need is not just a deficiency to be answered” but a process of path definition in collaboration with other services and territorial communities. In this sense, it is important to overcome the fragmentation of interventions and to encourage the cohesion and development of formalized and stable networks—which can also be flexible and adapt to continuous change—that guarantee a shared caring environment and continuity of interventions. Consequently, the form and quality of the networks, how they intervene and cope with difficulties, become nodes to be analyzed in order to better understand the real situation.

Our contribution has the privilege to be one of the first to focus on the functioning of networks for the reception and care of forced migrants with mental health issues—as no in-depth knowledge on this issue seems to be in the literature—and to shed light on this topic. In addition, the paper has the advantage of providing practical tools, suggested directly by the professionals, that can be immediately put in place to improve the network of services (e.g., the development of a shared medical record, the establishment of case managers, the implementation of a platform on which it is possible to share the information about the personal history of the beneficiaries, the institution of a research center).

These recommendations, however, may not always be easy to implement, so it would be desirable to activate in parallel training/accompaniment courses that can help professionals and organizations develop specific network skills in this field [57]. These kinds of interventions require specific skills, such as psychological skills. Consequently, they should be entrusted to professionals who can work to improve communication and cooperation between different actors and thus contribute to the strengthening of the service network [58].

## Figures and Tables

**Table 1:** Services areas.

Areas	Types of Organizations	Tasks
Health & psychological well-being area	Hospital (psychiatric department), associations, territorial services	Health mental care
Reception area	Reception centers	Housing, language courses, financial support for food, medical care support for integration, job orientation
Family area	Social cooperative, municipality	Support to families and minors
Legal area	Municipal legal office	Legal assistance

**Table 2:** Participants.

Participants	Sex	Age	Professional Role	Services Area
1	F	53	Coordinator	Family area
2	M	47	Coordinator	Family area
3	F	34	Social workers	Family area
4	M	37	Psychologist	Family area
5	F	29	Practitioners	Family area
6	M	48	Psychotherapist	Health & psychological well-being area
7	M	55	Psychotherapist	Health & psychological well-being area
8	M	43	Psychotherapist	Health & psychological well-being area
9	F	39	Psychotherapist	Health & psychological well-being area
10	M	59	Psychiatrists	Health & psychological well-being area
11	M	65	Psychiatrists	Health & psychological well-being area
12	F	35	Social workers	Health & psychological well-being area
13	F	28	Cultural mediators	Health & psychological well-being area
14	F	29	Legal practitioners	Legal area
15	F	34	Legal practitioners	Legal area
16	F	33	Legal practitioners	Legal area
17	M	42	Legal practitioners	Legal area
18	F	45	Cultural mediators	Legal area
19	F	38	Cultural mediators	Reception area
20	M	56	Coordinator	Reception area
21	M	49	Coordinator	Reception area
22	M	43	Cultural mediators	Reception area
23	F	35	Practitioners	Reception area
24	F	37	Practitioners	Reception area

**Table 3:** Superordinate and subordinate themes.

Superordinate Themes	Subordinate Themes	Quotes
Weaknesses of the network of services	1. Hostile social context	*“I perceived, as a professional who’s working in this area, a greater hostility from the citizen.”*
	2. Structural lack of resources	*“From my point of view, there is still a great hole in traditional psychiatry institutions.”*
	3. Negative emotions	*“That aggressiveness and that discouragement feeling could affect the operators’ integrity and institutions’ themselves.”*
	4. Organizational and management difficulties	*“What we perceive is a bit of stress in doing these things; on the other hand, we need more time and a less rigorous agenda.”*
	5. Institutional rigidity	*“I noticed this kind of closure on the part of some institutions. There was a tendency to use one’s own standard, one’s own model of thinking.”*
	6. Insufficient communication between services and not-clear-roles	*“There is a lack of coordination and communication between horizontal networks, which should coordinate better to be able to manage cases properly and find the right solution.”*
	7. Fragmented post-migratory paths	*“We are aware that the reception process will never be linear and easy [..] the beneficiaries, then, are forced to repeat many times their story.”*
	8. Totalizing institutions	*“Most of them live in these facilities as a place of transit, where they wait for specific answers (i.e., their documents) without investing a lot.”*
Strengths of the network of services	1. Mental health care	*“What has really changed is how we accept and trait their psychic suffering in the different areas of response.”*
	2. Professionals’ interpersonal relationships	*“As a service, being in touch with these realities is fundamental. We are in contact with the structures that deal with mental health, and it is useful to understand the work practices that we are supporting. Then the exchange of view could be both unidirectional and bidirectional.”*
	3. Relationship with beneficiaries	*“I have to say that working with asylum-seekers is very satisfying.”*
	4. Acknowledgment and trust among services	*“I have to say that I have never felt mistrust from other professionals for the work we do at the legal support office.”*
Possibility of improving the quality of the network	1. Need to formalize the network	*“If you do an interview with a beneficiary, you must consider at least two or three networks because working with migrants always requires connection and synergy with colleagues. So, there is a need for services to formalize this requirement and have moments of coordination; it would help us a lot.”*
	2. Development of new tools	*“I don’t know if it is practicable or possible, but I think that it would be innovative if these people were given either a case manager or a single folder that collects all the documentation.”*
	3. Need to train and inform	*“Everyone’s role […] is also to spread competence, and likewise, the role of the local social service should be to get information and be involved. […] the goal must be to create a competent community in this field so that they also are on the territory.”*
	4. Promote a culture of hospitality	*“We need to find a system to make ourselves promoters, not so much in a specific and specialized, or super-specialized, environment, where we only go to refine things. But we should create a basic resonance that, in the long run, could also generate and that could also be proactive for the single user. Become promoters of a territorial culture a little more detailed, concrete and informed about what we do and what our beneficiaries require.”*

## Data Availability

The data presented in this study are available on request from the corresponding author. The data are not publicly available due to privacy issues.
